# Genetic mapping and transcriptomic characterization of a new fuzzless-tufted cottonseed mutant

**DOI:** 10.1093/g3journal/jkaa042

**Published:** 2020-12-24

**Authors:** Qian-Hao Zhu, Warwick Stiller, Philippe Moncuquet, Stuart Gordon, Yuman Yuan, Scott Barnes, Iain Wilson

**Affiliations:** 1 Black Mountain Laboratories, CSIRO Agriculture and Food, Canberra, Australian Capital Territory 2601, Australia; 2 CSIRO Agriculture and Food, Narrabri, NSW 2390, Australia; 3 CSIRO Agriculture and Food, Waurn Ponds, VIC 3216, Australia; 4 CSIRO Manufacturing, Waurn Ponds, VIC 3216, Australia

**Keywords:** fuzzless-tufted cottonseed, fiber mutant, genetic mapping, near-isogenetic line, RNA sequencing

## Abstract

Fiber mutants are unique and valuable resources for understanding the genetic and molecular mechanisms controlling initiation and development of cotton fibers that are extremely elongated single epidermal cells protruding from the seed coat of cottonseeds. In this study, we reported a new fuzzless-tufted cotton mutant (*Gossypium hirsutum*) and showed that fuzzless-tufted near-isogenic lines (NILs) had similar agronomic traits and a higher ginning efficiency compared to their recurrent parents with normal fuzzy seeds. Genetic analysis revealed that the mutant phenotype is determined by a single incomplete dominant locus, designated *N_5_*. The mutation was fine mapped to an approximately 250-kb interval containing 33 annotated genes using a combination of bulked segregant sequencing, SNP chip genotyping, and fine mapping. Comparative transcriptomic analysis using 0–6 days post-anthesis (dpa) ovules from NILs segregating for the phenotypes of fuzzless-tufted (mutant) and normal fuzzy cottonseeds (wild-type) uncovered candidate genes responsible for the mutant phenotype. It also revealed that the flanking region of the *N_5_* locus is enriched with differentially expressed genes (DEGs) between the mutant and wild-type. Several of those DEGs are members of the gene families with demonstrated roles in cell initiation and elongation, such as calcium-dependent protein kinase and expansin. The transcriptome landscape of the mutant was significantly reprogrammed in the 6 dpa ovules and, to a less extent, in the 0 dpa ovules, but not in the 2 and 4 dpa ovules. At both 0 and 6 dpa, the reprogrammed mutant transcriptome was mainly associated with cell wall modifications and transmembrane transportation, while transcription factor activity was significantly altered in the 6 dpa mutant ovules. These results imply a similar molecular basis for initiation of lint and fuzz fibers despite certain differences.

## Introduction

Cotton fibers are elongated epidermal cells of cottonseeds. Based on their length and attachment to the seed coat after ginning, cotton fibers are classified into two categories: long lint fibers and short fuzz fibers. Lint fibers can be easily pulled off from the seed coat during the ginning process, whereas fuzz fibers remain attached to the seed coat after ginning. In Upland cotton (*Gossypium hirsutum* L.), lint fibers differentiate before anthesis, initiate at the day of anthesis and can elongate to an average length of ∼30 mm, while fuzz fibers differentiate and initiate after initiation of lint fibers, usually at approximately 5 days post-anthesis (dpa), and can elongate only up to ∼5 mm (Stewart 1975; Zhang *et al.* 2007). Fuzz fibers have low economic value and compete with lint fibers for energy and carbon source during their development.

Cotton gin energy costs have risen more than other gin operating costs. Energy audits conducted in US cotton gins representing a range of capacities between 2009 and 2013 revealed the average participating saw gin used between 34 and 40 kWh to process one cotton bale, *i.e.* 227 kg (Funk and Hardin 2012; Funk *et al.* 2013). While gins have become larger and more efficient again in the nearly 10 years since those audits, energy costs as a proportion of gin’s operating costs have become larger and now exceed >20% of the total cost of ginning *cf.* 6–10% in the 1970s (Funk and Hardin 2012). Ginning of seed cotton without fuzz fibers, or fuzzless, generally consumes less power and causes less damage to the lint fibers as much less force is required to remove the lint from the seed coat than fuzzy-seeded cotton (Bechere *et al.* 2009, 2012). Breeding cotton cultivars with fuzzless, or naked, seeds are one of the goals of many cotton breeding programs. To this end, it is essential to have fuzzless seed germplasm and to understand the genetic and molecular mechanism controlling fuzz differentiation, initiation, and development.

Several natural and induced fuzzless cotton mutants have been reported in tetraploid cotton (*G. hirsutum* and *G. barbadense*). Genetic analysis has identified and assigned four fuzzless loci, including the dominant locus *N_1_* and the recessive loci *n_2_*, *n_3_*, and ${\text{n}}_{4}^{t}$ (for a review, see Fang *et al.* 2018). The *N_1_* mutation was first reported in 1927 (Kearney and Harrison 1927). Both homozygous *N_1_N_1_* and heterozygous *N_1_n_1_* genotypes produce completely fuzzless seeds that lack any tuft at the micropylar tip of the seed and have a significantly reduced lint percentage (Ware 1940; Turley and Kloth 2002; Turley *et al.* 2007). The *n_2_* mutation was documented in 1947 and also reported to have negative effect on lint percentage (Ware *et al.* 1947). The *N_1_* and *n_2_* mutations were assigned to a pair of homoeologous chromosomes, chromosome 12 (A12 of the A_t_ subgenome) and chromosome 26 (D12 of the D_t_ subgenome), respectively (Endrizzi and Ramsay 1980). The recessive *n_3_* locus was identified based on genetic analysis of the progeny derived from a cross between the *N_1_N_1_* and *n_2_n_2_* mutants as it is required for full expression of the fuzzless trait in accessions with a genotype of *n_2_n_2_* (Turley and Kloth 2002). Cotton plants with a genotype homozygous for all the three fuzzless loci, *i.e. N_1_N_1_n_2_n_2_n_3_n_3_*, produce seeds that lack both lint and fuzz fibers, or fiberless, implying a role of these loci in the development of both lint and fuzz fibers. The fourth reported fuzzless mutation, ${\text{n}}_{4}^{t}$, was induced by ethyl methanesulfonate (EMS) treatment. Seeds of the homozygous ${\text{n}}_{4}^{t}$$n_{4}^{t}$ mutant are partially naked with a tuft at the micropyle (Bechere *et al.* 2009, 2012). Unlike *N_1_* and *n_2_*, the ${\text{n}}_{4}^{t}$ mutation has been reported to have a less negative effect on lint percentage and is thus potentially more valuable in breeding fuzzless commercial cultivars (Bechere *et al.* 2009, 2012). The genomic locations of the *n_3_* and ${\text{n}}_{4}^{t}$ loci remain to be determined.

Thanks to the publication of cotton genomes and advances in high-throughput genotyping technologies, the gene responsible for the well characterized dominant *N_1_* mutation has been determined to be the A_t_-subgenome homoeolog of *MYB25-like* (*i.e. MYB25L_A12*). A noncoding transcript antisense to *MYB25L_A12* was found at its 3′ end, forming double-stranded RNA to produce small-interfering RNAs (siRNAs) to post-transcriptionally silence *MYB25L_A12*. The siRNAs generated from the *MYB25L_A12* locus are expected to be able to silence *MYB25L_D12* as well given the high-level sequence similarity between the two homoeologs. Consequently, the expression level of *MYB25-like* (both *MYB25L_A12* and *MYB25L_D12*) is very low in the developing ovules of the *N_1_* mutant (Wan *et al.* 2016). *MYB25-like* is a master transcription regulator involved in initiation and development of both lint and fuzz fibers as transgenic plants with silenced *MYB25-like* produce fiberless cotton seeds, *i.e.* lacking both lint and fuzz fibers (Walford *et al.* 2011). Genetic and gene expression analyses of *MYB25L_A12* and *MYB25L_D12* in cotton accessions with variable lint and fuzz phenotypes imply the identity of *n_2_* being a dysfunctional *MYB25L_D12* allele (Zhu *et al.* 2018). It has been proposed that initiation and development of lint and fuzz fibers are regulated tempospatially by the two homoeologs of *MYB25-like*, with lint and fuzz fibers being mainly determined by *MYB25L_A12* and *MYB25L_D12*, respectively (Zhu *et al.* 2018). In diploid *G. arboreum*, *MYB25-like* (*i.e. GaMYB25-like*) was also found to be associated with fuzz development; however, in contrast to its major role in relation to fuzz development in tetraploid cotton, the role of *GaMYB25-like* in fuzz development in *G. arboreum* seems to be not as significant as *GaGIR1* that has been proposed to be associated with fuzz development in *G. arboreum* based on investigations of several different fuzzless mutants (Feng *et al.* 2019; Liu *et al.* 2020). Identification of an additional *n_3_* locus underlying the fuzzless phenotype in the *n_2_n_2_* genetic background (Turley and Kloth 2002) and the finding of the same genes involved in development of both lint and fuzz fibers (Zhu *et al.* 2018) hint the complexity of the genetic and molecular mechanisms underlying fiber development in tetraploid cotton. Consistent with this notion, it has been suggested that the fuzzless phenotype in Pima S-7 (*G. barbadense*) is associated with at least five loci with the *n_2_* mutation being the major contributor (Zhu *et al.* 2018).

Forward and reverse genetics studies have generated a large body of information regarding genes and gene networks associated with cotton fiber development, but we still know little about why less than one-third of the ovule epidermal cells are differentiated to becoming lint fibers and what determines the fate of lint and fuzz fibers. To answer these questions and to enhance our understanding on the genetic networks responsible for initiation and development of fuzz fibers, and to provide tools for genetic improvement of lint fibers, it is essential to generate and characterize as many novel fiber mutants as possible. In this study, we reported a new fuzzless-tufted Upland cotton mutant with a lint percentage higher than that of the historic *N_1_* and *n_2_* mutants, investigated the genetics of the mutant and identified candidate genes responsible for the mutant phenotype. Comparative transcriptomic analysis uncovered biological and cellular functions associated with fuzz development and identified genes, with a known or unknown function in fiber development, potentially regulated by the fuzzless-tufted mutation.

## Materials and methods

### Plant materials, mapping populations, and field experiments

The fuzzless-tufted mutant (CSS386 or *N_5_*, *G. hirsutum*) with an unknown cause of the mutation is one of the germplasm in the CSIRO cotton collection. Sicala V-2 (conventional) and Sicot 71BRF (with insect and herbicide-resistant transgenes) are commercial cultivars developed by the CSIRO cotton breeding program. MCU-5 is an Upland cotton variety introduced from India for its Fusarium wilt resistance (Lopez-Lavalle *et al.* 2012). Sicala V-2 and MCU-5 were used to generate the F_2_ and backcross populations used in genetic analyses, SNP genotyping, and bulked segregant sequencing. Sicot 71BRF was used as recurrent parent for generation of insect and herbicide-resistant fuzzless-tufted introgression line to enable it to be grown in a commercial field to produce seed cotton for the experiment of ginning efficiency comparison.

**Figure 1:**
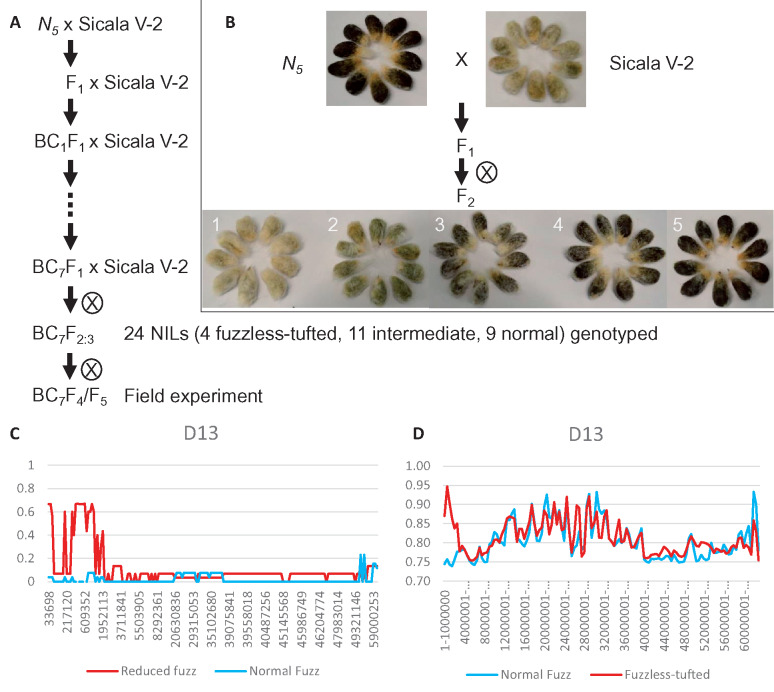
Development of near-isogenic lines (NILs) of the fuzzless-tufted mutant and genetic mapping by SNP genotyping and mapping-by-sequencing. (A) Development of NILs. In each generation, fuzzless-tufted seeds were used in further backcrosses. 24 BC_7_F_2:3_ NILs showing normal fuzz, intermediate fuzz, and fuzzless-tufted were genotyped using the Cotton SNP63K array. Selected homozygous normal fuzz and fuzzless-tufted NILs were used in RNA sequencing. (B) Seed phenotype of the fuzzless-tufted mutant, a normal fuzz *G. hirsutum* accession (Sicala V-2), and their F_2:3_ progeny. (C) SNP63K-array-based distribution of SNP frequency across chromosome D13. The graph was generated based on the frequency of individual SNPs. (D) Mapping-by-sequencing-based distribution of SNP frequency across chromosome D13. The graph was generated using a sliding window-based approach with a 1-Mbp window and 500-kb overlapping between the two adjacent windows.

The fuzzless-tufted trait has been introgressed into the genetic background of Sicala V-2 using seven backcrosses to develop near-isogenic lines (NILs) with the mutant or normal fuzzy seed phenotype (Figure 1A). A population of 24 BC_7_F_4_ and 16 BC_7_F_5_ fuzzless-tufted NILs derived from Sicala V-2, together with 50 BC_5_F_3_ or BC_6_F_4_ fuzzless NILs derived from Pima S-7 were compared to the three parents (CSS386, Sicala V-2, and Pima S-7) in field experiments at the Australian Cotton Research Institute (Narrabri, New South Wales, Australia), following standard commercial growing practices. A high-volume instrument (HVI) system was used to determine the fiber properties. Three populations were used in genetic mapping: an F_2_ population (NM) with 86 individuals from CSS386 (*N_5_*) × MCU-5, an F_2_ population (NS1) with 1800 individuals from CSS386 (*N_5_*) × Sicala V-2, and a BC_8_F_2_ population (NS) with 90 individuals from a cross between a fuzzless-tufted NIL line and Sicala V-2. These mapping populations were generated, grown, and phenotyped in a glasshouse in Canberra, Australia, at 28°C ± 2°C with natural lighting.

### Comparison of ginning efficiency

The trials to compare the energy required to gin the fuzzless-tufted (one of the BC_7_F_5_ NILs) and normal fuzzy (*G. hirsutum*, cv. Sicot 71BRF) seed cotton were conducted at CSIRO Manufacturing (Geelong, Victoria, Australia). Trials were done using a single stand, cut down (119 to 40 saws) industrial scale Continental Eagle saw gin stand with a Continental/Moss Gordin Galaxie feeder with a maximum output of just under two bales/hr (400 kg/h). Two gin saw motor speeds (slow: 35 Hz/500 rpm and fast: 43 Hz/600 rpm) were assessed. Speeds were modulated using an ABB variable frequency drive attached to the 50 kW gin saw motor. No lint cleaning was used. For each genotype, three ginning runs (50 kg/run) were conducted under each motor speed. Current (ampere) was measured using a clamp on ammeter to one of the three-phase lines to the gin motor. Data was recorded using a HOBO data-logger at a frequency of one Hz. Measured amperes were converted to power values (kW) using the peak voltage (415 V) for a single phase and assuming a power factor of one. The kilowatt hours (kWh) for each run were determined by multiplying the time to gin 50 kg seed cotton by the average kW value for the run. All ginning runs were carried out under warm, dry ambient conditions (18–23°C and 30–50% relative humidity) with the fiber moisture content <5.5%.

### CottonSNP63K array genotyping and bulked segregant sequencing

Four fuzzless-tufted, 11 intermediate and 9 normal fuzzy seed BC_7_F_2:3_ near-isogenic segregants were genotyped using the CottonSNP63K array as previously described (Hulse-Kemp *et al.* 2015; Zhu *et al.* 2016). To visualize the distribution of SNP frequency across chromosomes, the *N_5_*, Sicala V-2 and heterozygous alleles of each SNP were designed 1, 0, and 0.5, respectively, and plotted based on their chromosomal coordinates in the TM-1 reference genome (Zhang *et al.* 2015). Because only four fuzzless-tufted NILs were used, we combined them with the NILs with intermediate mutant phenotype when plotting the SNP frequency. The region with a SNP frequency of 0.75 or close to 0.75 in the fuzzless-tufted and intermediate pool, and a SNP frequency of 0 or close to 0 (assuming all these NILs having the Sicala V-2 background) in the normal fuzzy seed pool was considered to be the candidate region containing the gene(s) responsible for the mutation. For bulked segregant sequencing, bulked DNA pool was prepared using equal amount of DNA isolated from 26 fuzzless-tufted or 22 normal fuzzy seed F_2_ individuals. Each DNA pool was sequenced at ∼20× coverage of the *G. hirsutum* genome size. Short-reads mapping, SNP call, and plotting were performed as previously described (Zhu *et al.* 2018), except the reference genome, which was Sicala V-2 generated by mapping Sicala V-2 short-reads to TM-1 (Zhang *et al.* 2015).

### Fine genetic mapping

We first genotyped 1800 F_2_ individuals from CSS386 (*N_5_*) × Sicala V-2 using KASP (Kompetitive allele-specific PCR) markers AM3 and AM5 (Supplementary Table S1) located at the boundaries of the region identified based on SNP genotyping and bulked segregant sequencing. The analysis identified 276 segregants containing the region from the mutant. More KASP makers were then designed and used to genotype the identified 276 individuals. The results were used to construct a genetic linkage map and to perform QTL mapping using the software inclusive composite interval mapping (ICIM) (Li *et al.* 2007). The Kosambi mapping function was selected to convert a recombination frequency to genetic distance (cM). Linkage group and marker order were determined by using a logarithm of the odds (LOD) score of 15. QTL mapping was performed by using the ICIM-Add method of the ICIM software with 1000 permutation tests. Graphical representation was generated using MapChart (Voorrips 2002). KASP markers span the peak region were further used in genotyping of the NM and NS populations for co-segregation analysis.

### Kompetitive allele specific PCR genotyping

The KASP reaction was performed in an 8 μl volume, comprising 1 μl DNA (15 ng), 4 μl 2× KASP master mix (LGC Group), 0.11 μl primer mix (12 μM of each allele-specific primer and 30 μM of common primer) and 2.89 μl H_2_O. PCR cycling was performed on an Eppendof Mastercycler ep384 using the following program: hot-start at 95°C for 15′′, followed by 10 touchdown cycles (95°C for 20′′; touchdown at 65–57°C, 0.8°C per cycle, 60′′), and followed by 31 cycles of amplification at 94°C for 20′′ and 57°C for 60′′. Plates were read on the ViiA7 Real-Time PCR System (Life Technologies) at ambient temperature and analyzed using the Applied Biosystems software. If discriminating genotyping clusters had not formed after the above procedure, an additional three to six cycles of amplification were conducted and the plate was read and re-analyzed. KASP primers were designed based on polymorphic SNPs between the parental accessions (CSS386 and Sicala V-2) and are shown in Supplementary Table S1.

### DNA and RNA extraction

Genomic DNA used in SNP genotyping (CottonSNP63K array and KASP) and re-sequencing was extracted from young true leaves using a DNeasy Plant Mini kit (Qiagen) according to the manufacturer’s instructions or the cetyl trimethylammonium bromide (CTAB) method with home-made solutions. NanoDrop 1000 (Thermo Scientific) was used in the quantification of DNA concentration. DNA samples used in next-generation sequencing were also quantified using Qubit (Invitrogen) and checked for integrity by gel electrophoresis.

Total RNAs were extracted from 0, 1, 2, 3, 4, 5, and 6 dpa ovules of fuzzless-tufted and normal fuzzy seed NILs using the Maxwell RSC Instrument (Promega) with a Maxwell RSC Plant RNA Kit. RNA quality was checked using the Agilent 2100 Bioanalyzer (Agilent Technologies) and Qubit (Invitrogen). RNA samples from 0, 2, 4, to 6 dpa with an RNA integrity number (RIN) above 7.0 were used in RNA-sequencing, while samples from all seven time points were used in qRT-PCR analysis. Each sample was analyzed with three biological replicates.

### Transcriptome sequencing and identification of differentially expressed genes

RNA-sequencing was done using the paired-end (150 bp) configuration on an Illumina HiSeq 2000 instrument according to the manufacturer’s instructions (Illumina). Raw reads were first processed using Trimmomatic v0.39 (Bolger *et al.* 2014) to remove low-quality sequences and adaptors. The quality of trimmed FASTQ files was evaluated using FastQC v0.11.8. The clean reads were mapped to the TM-1 reference genome (Zhang *et al.* 2015) using Biokanga version 3.9.8 (https://github.com/csiro-crop-informatics/biokanga). The expression levels of annotated genes were estimated using Transcript Per Million (TPM) mapped reads. TPM and unnormalized reads were created using in-house python scripts and HTSeq count (Anders *et al*. 2015), respectively. Differentially expressed genes (DEGs) were defined using DESEq2 (Love *et al.* 2014). Genes with an adjust *P*-value < 0.05 and an absolute value of Log2 (fold-change) ≥ 1 were considered as DEGs. Gene ontology (GO) enrichment analysis was performed using agriGO v2.0 based on the default settings (Tian *et al.* 2017). Heatmaps were generated using ggplot2 of the *R* package (https://www.r-project.org/).

### Quantitative real-time reverse transcription PCR (qRT-PCR)

Total RNAs of 0 to 6 dpa whole ovules were used in the analysis of gene expression levels by qRT-PCR. Reverse transcription was performed using 2 µg of RQ1 DNase (Promega) treated total RNA, random hexamer, and SuperScript III reverse transcriptase (Invitrogen) in a 20 µl reaction. The first-strand cDNA reaction was diluted 10 folds and 4.6 µl of the diluted cDNA was then used as template in a 10-µl reaction with the FasterStart Universal SYBR Green Master Mix (ROX) (Roche). Negative controls with total RNA were performed to eliminate the possibility of genomic DNA contamination of the RNA samples. Relative gene expression levels were determined based on three biological replicates, each with three technical replicates using the 2^−ΔCt^ approach and tested by Student’s *t*-test for significance. The cotton ubiquitin gene (GenBank accession no. EU604080) was used as the reference gene for normalization. The reactions were run on the ViiA7 Real-Time PCR System (Life Technologies). The primers used in qRT-PCR are shown in Supplementary Table S1.

### Analysis of promoter *cis*-elements

One thousand base-pair sequence upstream of the start codon (ATG) of each gene was retrieved and submitted to the online database PLACE to identify *cis*-regulatory elements (Higo *et al.* 1999). The number of *cis*-elements that were found in the promoters of *Gh_D13G0096* and/or *Gh_D13G0098* was tallied separately for the regions of −1 to −500 bp (representing the core promoter) and −501 to −1000 bp.

### Gene cloning and analysis

Total RNAs (2 µg) of TM-1 and *N_5_* isolated from 5 dpa ovule using a Maxwell RSC Plant RNA Kit were reverse transcribed by oligo(dT) using the Superscript III first-strand synthesis system (Invitrogen) in a 20 µl reaction according to the manufacturer’s instruction. An aliquot of 2 µl cDNA reaction was then used in the amplification of the cDNAs using a Phusion^®^ High-Fidelity PCR Kit (New England Biolabs) with primers matching the start and end of *Gh_D13G0096* or *Gh_D13G0098*. PCR products were cut from gel, purified using the QIAquick Gel Extraction Kit, and ligated to the blunt DNA cloning vector of the Zero Blunt^TM^ PCR cloning kit (Invitrogen). Ten clones were sequenced to deduce the cDNA sequence of each gene.

### Data availability

All RNA-sequencing file and metadata information can be found at https://doi.org/10.25919/5ee196cce855a.

Supplementary material is available at figshare DOI: https://doi.org/10.25387/g3.13345268.

## Results

### Characterization of the fuzzless-tufted cottonseed mutant

The mutant line (CSS386) used in this study produces seeds that are fuzzless but with a tuft grown on the micropylar end (Figure 1B). Compared to the recurrent parent Sicala V-2, a conventional commercial cotton cultivar used previously in Australia cotton production, the mutant has a lower lint percentage and poorer fiber quality. After seven backcrosses, lint yield, lint percentage, and fiber quality of the advanced fuzzless-tufted NILs were equivalent to or even better than that of the recurrent parent. Compared to the fuzzless NILs derived from Pima S-7 x Sicala V-2, in which the fuzzless trait is determined by a recessive mutation, the advanced fuzzless-tufted NILs also had a higher lint yield and lint percentage although with shorter fibers (Table 1). The ginning efficiency of a fuzzless-tufted introgression line was compared with that of the recurrent parent Sicot 71BRF. It showed that the fuzzless-tufted introgression line used ∼10% and 14% less power under the slow and fast gin saw speed, respectively (Table 2). These results suggest that the fuzzless-tufted trait by itself has no negative effect on the performance of lint yield and fiber quality, and that it has the potential to save energy required for ginning seed cotton. The fuzzless-tufted trait is thus a valuable resource and is superior to the fuzzless trait from Pima S-7 for developing fuzzless commercial cotton cultivars.

**Table 1:** Comparison of lint yield, lint percentage and fiber quality between the fuzzless mutants, fuzzless NILs, and the recurrent parent

Genotype	No. of NILs	Lint yield (kg/ha)	Lint (%)	Fiber length (mm)	Uniformity (%)	Short fiber index	Fiber strength (g/tex)	Micronaire
Sicala V-2 (recurrent parent)		2534	40.65	30.73	84.50	8.90	33.10	4.25
CSS386 or *N5* (fuzzless- tufted mutant)		289	35.00	27.18	80.85	11.60	20.45	5.40
Fuzzless-tufted NILs derived from CSS386 (BC_7_F_4_)	24	2303 (1786–2615)	41.20 (39.40–42.75)	30.23 (28.96–32.77)	84.15 (81.20–86.25)	9.27 (8.10–11.10)	32.25 (30.85–34.15)	4.01 (3.15–4.52)
Fuzzless-tufted NILs derived from CSS386 (BC_7_F_5_)	16	2545 (2292–2738)	40.48 (39.20–41.70)	30.23 (29.46–31.24)	84.19 (82.30–85.63)	7.94 (6.63–9.50)	33.47 (32.20–34.63)	4.09 (3.53–4.53)
Pima S-7 (recessive fuzzless)		111	34.30	35.05	88.10	7.00	47.20	3.60
Fuzzless NILs-derived from Pima S-7 (BC_5_F_3_)	3	1771 (1682–1914)	38.13 (36.70–39.80)	31.49 (31.24–31.75)	86.76 (85.50–87.80)	7.78 (7.20–8.20)	31.90 (30.81–33.40)	4.41 (4.15–4.72)
Fuzzless NILs-derived from Pima S-7 (BC_6_F_4_)	47	2094 (1660–2547)	38.31 (36.83–41.10)	31.01 (29.21–32.51)	84.65 (83.10–86.13)	7.83 (6.70–9.70)	32.91 (30.57–35.70)	4.36 (3.83–4.87)

**Table 2:** Comparison of mean current trace statistics and power consumption between ginning the fuzzless-tufted and fuzzy seed cotton at two different motor speeds

Cotton seed phenotype	Seed cotton ginned/run (kg)	Motor speed of gin saw (Hz/rpm)	Current (ampere)	Power value (kW)	Ginning time (second)	Power consumed (kWh)
Fuzzless-tufted	50	35/500	14.91	6.19	255	0.438 (−10.06%)
Fuzzy	50	35/500	14.06	5.92	297	0.487
Fuzzless-tufted	50	43/600	15.37	6.38	267	0.472 (−14.03%)
Fuzzy	50	43/600	16.41	6.81	290	0.549

### Inheritance of the fuzzless-tufted cottonseed trait

To investigate the genetics underlying the fuzzless-tufted mutant trait, we generated two populations, one F_2_ population from CSS386 × MCU-5 (*i.e.* NM) and a BC_8_F_2_ (*i.e.* NS) population derived from a cross between a near-isogenic mutant line and the recurrent parent Sicala V-2. In both populations, F_2:3_ seeds were all tufted on the micropylar end but showed variable amount of fuzz on other parts of the seed coat. Based on the amount of fuzz, we separated the seeds into five groups, and considered groups 2 and 3 as intermediate and groups 4 and 5 as fuzzless (Figure 1B). For genetic analysis, segregants showing groups 2–5 seed phenotype were designated fuzzless-tufted. Using this criterion, the segregation ratio of fuzzless-tufted and fuzzy in both populations fits a single segregating locus model (Table 3). Due to the presence of intermediate mutant phenotypes, we propose that the fuzzless-tufted mutant trait is controlled by a single locus with incomplete dominance and designate the locus (or gene) responsible for the mutation *N_5_*.

**Table 3:** Genetic analysis of the fuzzless-tufted cottonseed trait

Population	Female	Male	Fuzzless-tufted	Fuzzy	*χ* ^2^_(3:1, 1=3.84)_	*P*-value
NM (F_2_)	CSS386 (*N_5_*)	MCU-5	62	24	0.42	0.51
NS (BC_8_F_2_)	CSS386 (*N_5_*)*-NIL*	Sicala V-2	64	26	0.72	0.39

### Identification of genetic region associated with the fuzzless-tufted cottonseed trait

Due to the nature of the incomplete dominance of the mutation, we reasoned that NILs showing the mutant or normal fuzzy seed phenotype would be the best choice for genetic mapping as their genetic background should have almost the same effect on the mutant phenotype. To this end, we introgressed the mutation into Sicala V-2 by seven backcrosses, and genotyped 4, 11, and 9 BC_7_F_2:3_ NILs showing fuzzless-tufted, intermediate, and normal fuzzy seed phenotype, respectively, using the CottonSNP63K array (Figure 1A). Of the ∼63 K SNPs, 5456 are polymorphic between the mutant and Sicala V-2, 4802 of them are distributed across the 26 cotton chromosomes (Supplementary Table S2). As expected, all chromosomes, except an ∼1.5 Mbp region on chromosome D13, of the fuzzless-tufted and intermediate NILs have been replaced by those from Sicala V-2 (Figures 1C and Supplementary Figure S1). The D13 region is thus where the mutation is most likely located. This was confirmed by bulked segregant sequencing of two DNA pools prepared using fuzzless-tufted or normal fuzzy seed segregants of a BC_8_F_2_ population (Figures 1D and Supplementary Figure S2).

### Fine mapping the genetic locus responsible for the fuzzless-tufted cottonseed trait

**Figure 2:**
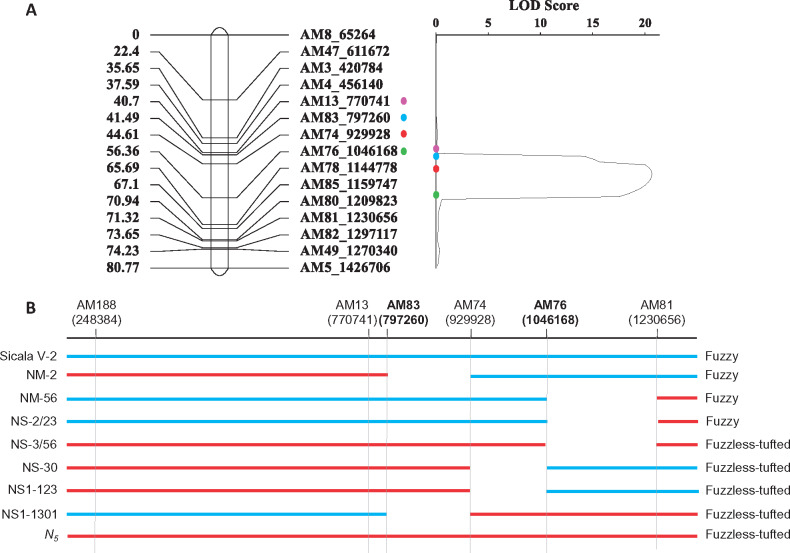
Fine mapping of the fuzzless-tufted mutation. (A) The fuzzless-tufted mutation was considered as a quantitative trait and mapped using the F_2_ segregants from *N_5_* × Sicala V-2 (the NS1 population). The IciMapping program was used in generating the linkage map and LOD score. (B) The genetic interval containing the fuzzless-tufted mutation was further confirmed using segregants from the NM (*N_5_* x MCU-5) and NS (a BC_8_ fuzzless-tufted NIL × Sicala V-2) populations.

To fine map the mutation responsible for the fuzzless-tufted trait, we developed an F_2_ population [NS1 = CSS386 (*N_5_*) × Sicala V-2] with 1800 individuals. We first genotyped all 1800 F_2_ individuals using two KASP markers (AM3 and AM5) located at the boundary of the D13 region to identify 276 segregants that showed recombination in the D13 region, *i.e.* the two markers had different origin (CSS386 or Sicala V-2). The identified segregants were then genotyped using another 13 KASP markers located between AM3 and AM5. Quantitative trait locus (QTL) analysis was done using the genotyping results and the phenotyping data of F_2:3_ seeds, which identified four markers (AM13, AM83, AM74, and AM76) to be closely linked with the mutant phenotype. The genetic interval defined by AM13 and AM76 is ∼275 kb (Figure 2A). These four markers and several flanking markers were then used to genotype the two populations (*i.e.* NM and NS) used in genetic mapping. Together, the genotyping and phenotyping results allowed us to further narrow down the causative region to an ∼249 kb region, between markers AM83 and AM76 (Figure 2B). We noticed that, in all three segregating populations, some heterozygous plants, *i.e.* with a genotype of *n_5_N_5_*, produced fuzzless-tufted seeds, but all plants with intermediate fiber phenotype were heterozygous for the *N_5_* locus, consistent with the nature of incomplete dominance of the mutation.

### Candidate genes underlying the fuzzless-tufted seed trait

**Figure 3:**
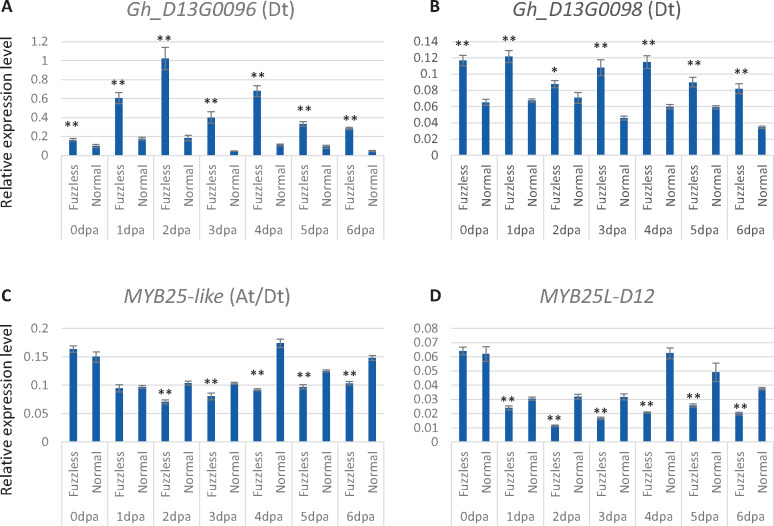
Comparison of the expression levels of the two differentially expressed genes at the fuzzless-tufted locus and that of *MYB25-like* between the *N_5_* mutant and its NIL showing normal fuzzy seeds in 0–6 dpa ovules. qRT-PCR was done using three biological replicates each with three technical replicates. Error bars represent standard deviations of the nine measurements. * and ** indicate significant difference at *p *<* *0.05 and *p *<* *0.01, respectively, determined by the Student’s *t*-test (two-tailed) based on three biological replicates each with three technical replicates.

The genetic interval between AM83 and AM76 has 33 annotated genes in the TM-1 genome (Zhang *et al.* 2015). To identify candidate gene(s) responsible for the fuzzless-tufted mutant phenotype, we did RNA-seq using RNAs isolated from 0, 2, 4, to 6 dpa ovules of the mutant and normal fuzzy seed NILs (hereafter, WT). Of the 33 genes located at the candidate region, 24 were expressed but only two (*Gh_D13G0096* and *Gh_D13G0098*) were differentially expressed between the mutant and WT in at least two of the four time points (Table 4), making them the best candidate genes contributing to fuzz initiation and development. Both *Gh_D13G0096* and *Gh_D13G0098* were upregulated in the mutant ovules, which was confirmed by qRT-PCR (Figure 3, A and B). In TM-1, these two genes are mainly expressed in ovule and fibers. *Gh_D13G0096* has a much higher expression level in 10–25 dpa ovules and 25 dpa fibers than in other tissues. *Gh_D13G0098* is mainly expressed in −3 to 10 dpa ovules (Supplementary Figure S3). *Gh_D13G0096* encodes a hypothetical protein without known conserved domain, while *Gh_D13G0098* is closely related to *cationic amino acid transporter 7* (*CAT7*) and *CAT6* of *Arabidopsis thaliana*, members of the amino acid-polyamine-choline (APC) transporter family.

**Table 4:** Expression changes [Log_2_(M/N)] of the annotated genes in the *N_5_* locus

Gene_ID	Start	End	Functional annotation	0 dpa	2 dpa	4 dpa	6 dpa
D13G0078	847797	849821	DC1 domain-containing protein	0.29	0.39	0.22	0.3
D13G0080	853400	854761		0.19	0.19	0.15	−0.1
D13G0081	861848	863553	Cytochrome b–c1 complex	−0.07	−0.15	0	0.17
D13G0082	865034	867466	Protein serine/threonine kinases	−0.92	1.17	−1.87	1.12
D13G0083	867592	868265	Protein of unknown function (DUF1118)	−0.43	0.04	0.27	−0.73
D13G0085	873770	877019	GroES-like zinc-binding alcohol dehydrogenase family protein	0.18	−0.1	−0.38	−0.35
D13G0086	877418	879560	Alpha/beta-Hydrolases superfamily protein	−0.08	0.06	−0.01	−0.15
D13G0087	881435	884262	Transducin/WD40 repeat-like superfamily protein	−0.03	0.08	−0.05	−0.07
D13G0088	887905	893110		0	0.05	0.03	0.03
D13G0089	894002	901520	Tetratricopeptide repeat (TPR)-like superfamily protein	−0.18	−0.01	−0.06	−0.16
D13G0090	902473	913155	Sec23/Sec24 protein transport family protein	0.02	0.18	0.26	0.24
D13G0091	918387	924132	DNAJ heat shock N-terminal domain-containing protein	−0.15	−0.12	−0.16	−0.07
D13G0092	927208	927526		−0.06	−0.38	0.21	−0.33
D13G0093	928840	932359	Nuclear transport factor 2 family protein	−0.22	0.58	0.58	−2.08
D13G0094	944433	947011	Double Clp-N motif-containing P-loop nucleoside triphosphate hydrolases superfamily protein	−0.21	0.31	−0.14	−0.26
D13G0095	959019	967550	Pentatricopeptide repeat (PPR) superfamily protein	−0.02	0.13	0.07	0.05
D13G0096	971457	972211		1.96^a^	2.56^a^	3.23^a^	2.72^a^
D13G0098	987476	990611	Cationic amino acid transporter 7	0.96	1.09^a^	0.85	1.11^a^
D13G0101	999015	1002839	Translocon at the outer envelope membrane of chloroplasts 159	0.06	0.22	0.12	0.04
D13G0102	1008601	1012317	Translocon at the outer envelope membrane of chloroplasts 159	0.12	0.03	−0.03	0.06
D13G0103	1018928	1022750	ACT domain-containing protein	−0.14	−0.19	−0.07	−0.13
D13G0104	1024398	1025657	Trichome birefringence-like 36	−0.16	−0.08	−0.39	−0.52
D13G0105	1029310	1032443	Regulatory particle triple-A ATPase 3	−0.04	0.12	0.1	0.13
D13G0106	1033701	1042658		−0.35	0.42	−1.15	−1.74

a Significantly differentially expressed between the mutant and WT.

No genomic sequence difference was found between the mutant and TM-1 in both *Gh_D13G0096* and *Gh_D13G0098*. For *Gh_D13G0098*, its flanking 1-kb regions were also identical between TM-1 and the mutant. Compared to TM-1, the mutant has three indels in the promoter of *Gh_D13G0096* (a single base insert between positions 215 and 216 counting from the start codon) or downstream of the gene (a single base insert between positions 653 and 654, and a deletion of two bases between positions 807 and 808, counting from the stop codon) (Supplementary Figure S4). For both genes, it is unclear what causes their up-regulation in the mutant, although for *Gh_D13G0096* the possible contribution of the three indels located at up- or down-stream the coding sequence could not be ruled out.

Next, we cloned cDNAs of both genes. Consistent with the annotation, no difference was found in *Gh_D13G0096* between TM-1 and the mutant. But in *Gh_D13G0098*, we found that the annotated first intron (71 bp) is retained in the cDNAs of both TM-1 and the mutant, which introduces a pre-mature stop codon at the beginning of the second exon. As a result, the cloned cDNAs contain two open reading frames, ORF1 and ORF2 that are equivalent to *Ghir_D13G001110* and *Ghir_D13G001100*, respectively, in the TM-1 reference genome reported by Huazhong Agricultural University (Gh_HAU; Wang *et al.* 2019). ORF1 is identical to *Ghir_D13G001110* but ORF2 and *Ghir_D13G001100* have different starts and ends (Supplementary Figures S5 and S6A). Despite the difference observed between the annotated *Gh_D13G0098* and its cloned cDNAs, all 10 cDNA clones from TM-1 or the mutant were identical, *i.e.* with the retained 1st intron, implying that the alternative splicing event is unlikely to be the cause of the mutation.

The annotation difference observed between *Gh_D13G0098* (Gh_NAU) and its counterparts in Gh_HAU prompted us to compare *Gh_D13G0096* and *Gh_D13G0098* as well as their flanking genes with their counterparts in the five published TM-1 genomes (Zhang *et al.* 2015; Hu *et al.* 2019; Wang *et al.* 2019; Chen *et al.* 2020; Huang *et al.* 2020). For *Gh_D13G0098* (Gh_NAU), except Gh_HAU (Wang *et al.* 2019), an identically annotated gene was found in Gh_ZJU (Hu *et al.* 2019), Gh_WHU (Huang *et al.* 2020), and Gh_JGI (Chen *et al.* 2020). *Gh_D13G0096* and its counterparts were found in all five TM-1 genomes, but interestingly two and three tandem copies of the gene were annotated in the Gh_WHU and Gh_JGI versions of the TM-1 genome (Supplementary Figure S6B; Huang *et al.* 2020; Chen *et al.* 2020). Most genes flanking *Gh_D13G0096* and *Gh_D13G0098* have the same annotation in different versions of the TM-1 genome. The discrepancy in annotation of *Gh_D13G0096* and a few other genes in the *N_5_* locus implies the difficulty involved in assembly and annotation of the region possibly due to copy number variation and/or other unknown structural variation(s). We re-sequenced the mutant genome using the second-generation sequencing technology, but it is currently impossible to know if the mutant is different from TM-1 in genome structure and/or gene copy number at the *N_5_* locus.

We also checked coding sequence variations for all other annotated genes at the *N_5_* locus. Nucleotide variations (SNPs and indels) causing amino acid changes in the *N_5_* mutant were found only in two genes (*Gh_D13G0089* and *Gh_D13G0090*) that were not differentially expressed between the mutant and WT. Two and one amino acid substitution was observed in Gh_D13D0089 and Gh_D13G0090, respectively (Supplementary Figure S7).

### The flanking region of the *N_5_* locus is enriched with differentially expressed genes

**Figure 4:**
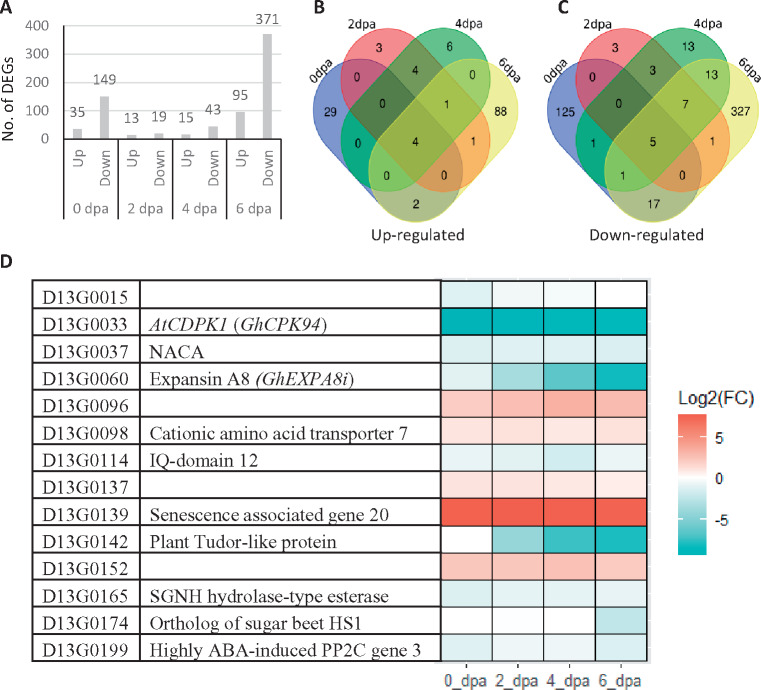
Transcriptomic comparison of the fuzzless-tufted mutant and its NILs showing normal fuzzy seeds. (A) Number of up- and down-regulated genes at 0–6 dpa ovules. (B) Overlapping of up-regulated genes. (C) Overlapping of down-regulated genes. (D) Expression changes of the differentially expressed genes at the *N_5_* locus and its 10-Mbp flanking regions at 0–6 dpa ovules.

Overall, transcriptome analysis identified 740, with 649 non-redundant, genes differentially expressed between the mutant and WT in 0–6 dpa ovules. Of which, 158 (133 non-redundant) and 582 (516 non-redundant) were up- and down-regulated in the mutant, respectively (Supplementary Table S3). The majority DEGs were found at 6 dpa (63%), followed by 0 (25%), 4 (8%), and 2 (4%) dpa (Figure 4A). Most DEGs (90%) were unique to a single time point and only 46, 9, and 9 nonredundant DEGs were overlapping between 2, 3, and all 4 time points, respectively (Figure 4, B and C). Of the 649 non-redundant DEGs, 29 were on chromosome D13 and 14 of the 29 D13 DEGs were located at the *N_5_* locus and its 10-Mbp flanking regions (Figure 4D), including 5 (*Gh_D13G0033, Gh_D13G0037, Gh_D13G0096, Gh_D13G0139*, and *Gh_D13G0152*) of the nine nonredundant genes that were differentially expressed at all four time points, and 4 (*Gh_D13G0060*, *Gh_D13G0098*, *Gh_D13G0114*, and *Gh_D13G0199*) of the 46 nonredundant genes that were differentially expressed at two of the four time points. Amongst them were two (*D13G0033* and *D13G0114*) related to Ca^2+^ signaling and one (*D13G0060*) being a member of the expansin family. *Gh_D13G0033* or *GhCPK94* (Gao *et al.* 2018) is closely related to *calcium dependent protein kinase 1* (*AtCDPK1*) of *A. thaliana* and was significantly down-regulated at all four time points. Similarly, *D13G0114*, an IQ-domain containing gene, was also down-regulated at all four time points, particularly at the 4 dpa mutant ovules. In WT ovule, the expression level of *Gh_D13G0060* or *GhEXPA8i* (Lv *et al.* 2020), a homolog of *A. thaliana Expansin A8* (*AtEXPA8*), increased almost 30 times in ovules from 0 dpa (∼10 TPM) to 6 dpa (∼300 TPM), with a surge observed from 4 dpa (∼90 TPM) to 6 dpa. By contrast, the expression level of *Gh_D13G0060* was very low (<6 TPM) in the mutant ovules, particularly in the 4 and 6 dpa ovules, consequently a significant down-regulation of the gene was observed in the 4 and 6 dpa mutant ovules (Supplementary Table S3, Figure 4D). Intriguingly, the expression level of *Gh_D13G0139*, a homolog of *Arabidopsis senescence associated gene 20* (*AtSAG20*), was quite low (∼40–70 TPM) in the 0–6 dpa WT ovules, but its expression level was extremely high (6600–13,000 TPM) in the mutant ovules, consequently, *Gh_D13G0139* was the most significantly up-regulated gene at all four time points in the mutant ovules (Figure 4D).

Enrichment of DEGs at the *N_5_* locus and its flanking regions indicates the existence of a cluster of co-regulated and functionally related genes associated with the mutant phenotype. A similar phenomenon has been reported for the genomic region with the *Ligon lintless-2* mutation (Patel *et al.* 2020). One possibility for co-regulation of functionally related genes is the presence of shared *cis*-regulatory elements in their promoters. Indeed, a number of *cis*-regulatory elements were found to be common in the promoters of these co-regulated genes (Supplementary Table S4). Alternatively, these co-regulated genes may belong to a large chromosomal domain regulated by the same chromatin conformation (Zhan *et al*. 2006).

### Transcriptome reprogramming in the mutant

**Figure 5:**
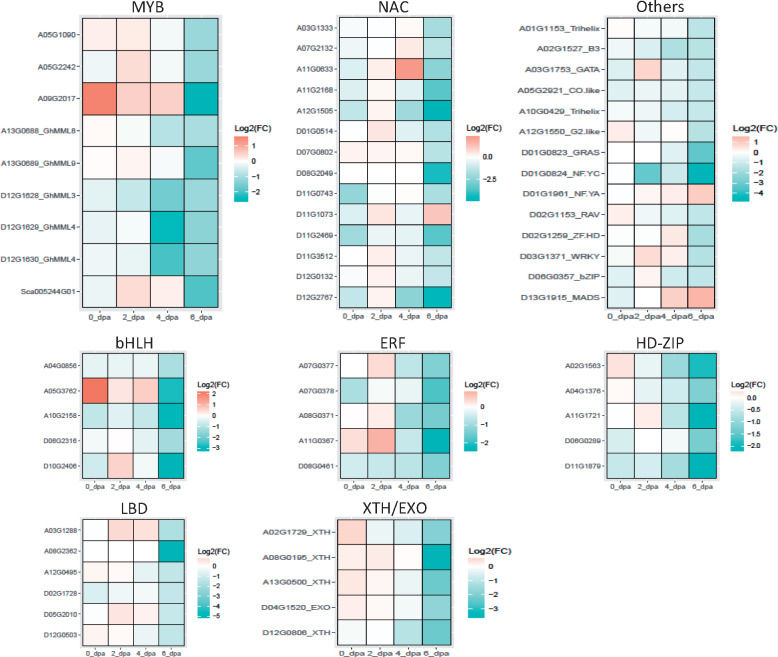
Expression change [Log_2_(mutant/normal)] of the transcription factors that were differentially expressed between the mutant and fuzzy seed NILs in the 6 dpa ovules. Three (*D11G1073*_NAC, *D01G1961*_NF.YA, and *D13G1915*_MADS) were up-regulated and all others were down-regulated in the 6 dpa ovules of the mutant.

To investigate the biological function of the DEGs, we searched enriched gene ontology (GO) terms for the up- or down-regulated DEGs identified at each time point. For the 35 and 13 upregulated DEGs identified at 0 and 2 dpa, respectively, no significantly enriched GO term was found. The 15 upregulated DEGs at 4 dpa were enriched for a single GO term, multi-organism process. For the 95 up-regulated DEGs identified at 6 dpa, enriched GO terms included cell wall modification, single-organism cellular process, and ion transmembrane transport (Supplementary Table S5). For the 149, 19, 43, and 371 down-regulated DEGs identified at 0, 2, 4, and 6 dpa ovules, respectively, no significantly enriched biological process GO term was found for the 2 and 4 dpa DEGs. At 0 dpa, the down-regulated DEGs were enriched with genes related to cellular responses to multiple stimuli, including lipid, oxygen-containing compound, and abscisic acid (ABA). The down-regulated DEGs identified at 6 dpa were not only enriched with genes related to responses to ABA, oxidative reaction, and lipid metabolism, but also with genes responses to water deprivation, ethylene (ET), and jasmonic acid (JA). Regarding molecular function, the down-regulated DEGs found at 0 dpa were enriched with genes involved in transmembrane transporter activity, such as nitrate transporter, ABC-2 type transporter, and sodium/calcium exchanger, while at 6 dpa the down-regulated DEGs were enriched with genes related to transcription factor activity. Only three transcription factors (TFs) were up-regulated in the mutant ovules at 6 dpa, but a total of 53 TFs, including multiple members of the MYB, NAC, bHLH, ERF, HD-ZIP, and LBD families, showed a significantly lower expression level in the mutant ovules than in the ovules of the fuzzy seed NILs (Figure 5). Of the 10 members of the R2R3-MYB subgroup *MYBMIXTA-like*, or *MML*, 4 were significantly down-regulated in the 6 dpa mutant ovules, including *MYB25-like* (*GhMML3*), *GhMML4*, *GhMML8*, and *GhMML9* (Figures 3C and 5). *MYB25L_D12* that has been shown to be the major gene determining fuzz development in *G. barbadense* (Zhu *et al.* 2018) had a lower expression level in the 1–6 dpa mutant ovules (Figure 3D). Other genes reported to be related to fiber initiation and elongation, such as *GhHOX1*, *GhHOX2*, *GhHOX3*, *GhVIN1*, *GhJAZ2*, and *GhCaM7* all were not differentially expressed between the mutant and WT, although *GhHD1* was marginally significantly down-regulated in the 6 dpa mutant ovules.

The down-regulated DEGs identified at 0 and 6 dpa have a cellular functionality related to cell periphery and cell wall, respectively (Supplementary Table S5). In addition to *GhEXPA8i* (*Gh_D13G0060*), several genes encoding xyloglucan endotransglucosylase/hydrolase (XTH) and exordium (EXO) were significantly down-regulated in the 6 dpa mutant ovules. Among the up-regulated DEGs found in the 6 dpa mutant ovules were genes encoding plasma membrane intrinsic proteins or involved in potassium transportation (Supplementary Table S3). The functions of all these genes in cotton fiber initiation remain to be explored.

Together, these results imply that the transcriptome landscape of the mutant is relatively similar to that of WT in the 2 and 4 dpa ovules, but has reprogrammed in the 0 and 6 dpa ovules, particularly at 6 dpa when fuzz fibers start to initiate and/or development, for genes involved in transmembrane transportation, cell wall modifications, and TF activities.

## Discussion

Numerous fuzzless cotton mutants with a variable amount of lint fibers have been reported. The historic dominant *N_1_* mutant has a very low lint percentage and consequently significant penalty on lint yield. From a breeding perspective, such a mutant has little utility, although it is valuable for understanding fiber initiation and development. But some EMS induced fuzzless mutants were reported to have comparable lint percentage compared to the cultivar from which the mutant was induced, and they have been used in breeding programs to develop fuzzless genotypes as the fuzzless trait has the potential to reduce short fiber content and to improve both ginning quality and efficiency (Bechere *et al.* 2009, 2012; Hendon *et al.* 2019). The fuzzless-tufted mutant reported in this study has a lint percentage of ∼35%. After several backcrosses, lint yield, lint percentage, and fiber quality of the fuzzless-tufted NILs were similar to or higher than that of the recurrent parent (Table 1). The commercial case for fuzzless seed cotton cultivar also lies in improved ginning efficiency. Comparison of a fuzzless-tufted introgression line with its recurrent parent (Sicot 71BRF) illustrated the extent of the benefit (Table 2). These results demonstrate the breeding value of the fuzzless-tufted mutant. To facilitate introgressing the trait into elite commercial cultivars and to understand the molecular mechanism underlying the mutant phenotype, we genetically characterized the mutant, determined the mutant phenotype to be controlled by a single genetic locus (*N_5_*) with incomplete dominance, and mapped the mutant locus to a chromosomal location that has not previously been associated with fiber development (Figure 2). Two genes, *Gh_D13G0096* and *Gh_D13G0098*, were considered to be the candidates related to fuzz initiation and development based on their location in the genetic interval containing the fuzzless-tufted mutation and their differential expression profile in the developing ovules of the mutant and WT (Table 4). We are currently unsure about the nature of the mutations in these two candidate genes that causes their up-regulation in the mutant, as it is complicated by the variable copy numbers of *Gh_D13G0096* annotated in a different version of the same TM-1 genome and the alternative splicing events of *Gh_D13G0098* (Supplementary Figure S6B).

Up-regulation of *Gh_D13G0096* and *Gh_D13G0098* in the mutant suggest that they might be repressors of fuzz initiation and/or development. The function of *Gh_D13G0096* cannot be inferred due to lack of characterized homologous gene in other plants, but given its expression pattern in various tissues of TM-1 (Supplementary Figure S3), *Gh_D13G0096* might play a more important role in fiber elongation or seed development than in fuzz initiation. *Gh_D13G0098* encodes an amino acid-polyamine-choline (APC) transporter. Amino acids are indispensable for cell biology and plant development, and act as regulators of metabolic pathways. Their translocation within plants and transport across intracellular membranes are mediated by multiple families of amino acid transporters, including APC transporters. APC transporters have been identified in almost all organisms, implying their functional conversion and fundamental roles in cell biology (Wipf *et al.* 2002). The *Arabidopsis* genome contains 14 APC-type transporters, including 9 cationic amino acid transporters (AtCAT1 to AtCAT9; Su *et al.* 2004). *Gh_D13G0098* is closely related to *AtCAT7* and *AtCAT6*. The physiological and molecular function of AtCAT7 remains to be investigated but it has been suggested to act as a compatible solute transporter because the expression level of *AtCAT7* is low under standard growth conditions but induced by treatment of NaCl (Su *et al.* 2004). *AtCAT6* is preferentially expressed in sink tissues, including the outer epidermal cell layer of embryo, and is localized primarily in the plasma membranes. AtCAT6 transports both cationic and large, neutral amino acids (Hammes *et al.* 2006). Another two members of the *Arabidopsis* APC transporter family, AtCAT2 and AtCAT4, are tonoplast localized with AtCAT2 being important for regulation of the concentration of leaf amino acids (Yang *et al.* 2014). It is thus of interest to investigate the cellular localization and substrate specificity of Gh_D13G0098. We speculate that up-regulation of *Gh_D13G0098* might have altered the homeostasis of certain amino acids and/or their analogs in the epidermal cells, leading to failure of the epidermal cells to be initiated as fuzz fibers via a gene network regulating Ca^2+^-signaling, reactive oxygen species signaling, and cell wall loosening and transportation.

Calcium (Ca^2+^) is an essential macronutrient in plants. It also serves as an important secondary messenger in plant development and has been shown to regulate diverse physiological processes and organ development, such as pollen tube growth and root hair elongation (Brost *et al.* 2019; Zheng *et al.* 2019). Comparative transcriptome analysis in cotton has suggested the importance of Ca^2+^-signaling in fiber initiation as many down-regulated genes at the fiber initiation stage in the fiberless mutant are involved in calcium-mediated signal transduction pathways (Padmalatha *et al.* 2012). The essentialness of Ca^2+^ in fiber initiation has been further demonstrated by *in vitro* ovule culture experiments, in which no fiber initiation was observed on −1 dpa ovules cultured for 72 h on the medium without Ca^2+^ and fiber initiation could be reinitiated by supplying Ca^2+^ (Zhang *et al.* 2017a). Plants have several classes of Ca^2+^ sensors recognizing specific Ca^2+^ signals and transducing them into downstream effects, including calmodulins (CaMs), calmodulin-like proteins (CMLs), calcineurin B-like proteins (CBLs), and calcium-dependent protein kinases (CDPKs or CPKs) (Shi *et al.* 2018). In cotton, early studies have found that genes encoding different types of Ca^2+^ sensors are among those that are preferentially expressed in elongating fibers, suggesting an important role of Ca^2+^-mediated signal transduction in fiber elongation (Gao *et al.* 2007; Huang *et al.* 2008). A recent study showed that, in cotton accession with normal fiber, the expression level of *GhCaM7* gradually increases in 0–15 dpa ovules and correlates with the Ca^2+^ influx rate from the extracellular to the intracellular environment at the fiber tips. By contrast, fiberless accessions have a much lower expression level of *GhCaM7* in 0 dpa ovules, implying a role of *GhCaM7* in not only fiber elongation but also fiber initiation, which is supported by transgenic cottons with overexpressed or down-regulated expression level of *GhCaM7* (Tang *et al.* 2014). In this study, we found a CPK-encoding gene *D13G0033* (or *GhCPK94*; Gao *et al.* 2018) in the flanking region of the *N_5_* locus. The expression level of *D13G0033* in the 0 - 6 dpa ovules was constantly significantly lower in the mutant than in the WT (Figure 4), suggesting that Ca^2+^ influx and/or signaling is compromised in the mutant, consistent with a positive role of Ca^2+^ in fiber initiation. In addition, a gene (*D13G0114*) encoding an IQ-domain-containing protein that mediates calmodulin (Bahler and Rhoads 2002), an archetypal Ca^2+^-sensor, was found in the flanking region of the *N_5_* locus. Together, these findings suggest that multiple Ca^2+^-signaling pathways could have been affected by the fuzzless-tufted mutation.

Expansins are cell-wall loosening proteins involved in cell enlargement by local sliding of wall polymers without hydrolytic breakage of major structural components of the cell wall. They are involved in almost all physiological processes during plant development (Marowa *et al.* 2016). Several expansin genes preferentially expressed in developing cotton fibers have been identified (Harmer *et al.* 2002; Li *et al.* 2016; Lv *et al.* 2020). For instance, *GbEXPATR* is an atypical α-expansin that lacks domain 2 and is expressed in developing fibers of *G. barbadense* accessions but not in those of *G. hirsutum* accessions, implying a role of *GbEXPATR* in fiber elongation as *G. barbadense* fibers are much longer than *G. hirsutum* ones. Supporting this was the observation that transgenic plants overexpressing *GbEXPATR* had longer, stronger, and finer fibers (Li *et al.* 2016). Longer fibers were also observed in transgenic cotton plants overexpressing another α-expansin gene, *GhEXPA8* (Bajwa *et al.* 2015). In WT ovules, the expression level of *D13G0060* or *GhEXPA8i*, one of the 10 *GhEXPA8* genes, gradually increases from 0 to 7 dpa, significantly decreases after 10 dpa and remains low afterwards (this study and Lv *et al.* 2020). The time period with the highest expression level of *D13G0060* in ovules co-occurs with the time of fuzz initiation (Stewart 1975; Zhang *et al.* 2007). The expression level of *D13G0060* was down-regulated in 0–6 dpa ovules in the fuzzless-tufted mutant, particularly in 6 and 4 dpa mutant ovules (Figure 4D). Given the expression pattern of this gene in developing ovules and fibers, and the observation of a very low expression level in the 4 and 6 dpa mutant ovules (Supplementary Table S3), we propose that *D13G0060* or *GhEXPA8i* may have a specific role in initiation of fuzz fibers.

One interesting finding from the transcriptome analysis is the up-regulation of a senescence associated gene (*D13G0139*) located in the flanking region of the *N_5_* locus. *D13G0139* is the most significantly up-regulated gene at all four time points in the mutant ovules (Supplementary Table S3). *D13G0139* is homologous to *Arabidopsis AtSAG20*, a gene that has not been functionally characterized. Despite it is currently unable to speculate if *D13G0139* play a role in fiber cell differentiation and initiation, the expression level of another senescence associated gene, *AtSAG21*, has been reported to play a role in root hair elongation as *Arabidopsis* plants overexpressing *AtSAG21* have a higher proportion of longer root hairs whereas the root hairs of the plants with down-regulated *AtSAG21* are shorter. And *AtSAG21* was found to be located to mitochondria (Salleh *et al.* 2012). As the expression level of *AtSAG21* was up-regulated in response to oxidants, it was proposed that *AtSAG21* might function as a co-factor maintaining the stability of the mitochondria proteins involved in production and/or signaling of reactive oxygen species (Salleh *et al.* 2012). Up-regulation of *D13G0139* in the mutant ovules suggests it being a negative regulator of fuzz initiation, different from the positive role of *AtSAG21* in root hair initiation and elongation. The role of *D13G0139* in fiber initiation and elongation warrants further investigation.

Based on the number of identified DEGs and enriched GO terms at each time point, it seems that the effect of the *N_5_* mutation is mainly at 0 and 6 dpa, particularly at 6 dpa, consistent with the fuzzless mutant phenotype, as fuzz fibers are believed to be initiated at 5–10 dpa (Stewart 1975; Zhang *et al.* 2007). Compared to the published transcriptome studies based on fiberless (lintless and fuzzless) mutants (Padmalatha *et al.* 2012; Wan *et al.* 2014), our transcriptome analysis, using fuzzless mutant, should work better in the identification of genes related to fuzz development. Indeed, many genes that have not been previously associated with fiber development were uncovered to have a potential role in fuzz development, including many TFs and genes encoding proteins involved in cell wall extensibility, such as *XTH* and *EXO*. The primary cell walls of plants consists of cellulose microfibrils tethered by pectins and xyloglucans. XTH functions as cell wall loosening enzymes by cleaving the links between xyloglucans and cellulose microfibrils (Park and Cosgrove 2015). Overexpressing *GhXTH1* was able to increase fiber length by 15–20% (Lee *et al.* 2010). EXO has been shown to be required for leaf cell expansion and a potential negative regulator of cell division (Farrar *et al.* 2003; Schroder *et al.* 2009). Significant down-regulation of *XTH* and *EXO* genes observed at 6 dpa in the fuzzless-tufted mutant implies an association of these genes with initiation of fuzz fibers (Figure 4). Furthermore, it has been suggested that xyloglucans tend to intertwin with cellulose microfibrils at the sites where α-expansins act to loosen cell wall (Park and Cosgrove 2015), suggesting that initiation of fuzz fibers may require cell wall loosening coordinated by XTH and expansins.

As elongated epidermal cells, the formation of lint and fuzz fibers undergoes cell specification, initiation, and elongation three phases. We know almost nothing about the biological process specifying the fate of fiber cells, although auxin accumulation in fiber-to-be facilitated by polarized transport of auxin from neighboring non-fiber epidermal cells seems to be crucial for the fiber-to-be becoming a fiber initial (Zhang *et al.* 2017b; Zeng *et al.* 2019). We are unable to infer if the *N_5_* mutation has a role in specification of epidermal cells to becoming fuzz fibers. Once the fate of fuzz fiber cells being determined, localized, and/or polarized cell wall loosening may be a prerequisite for protrusion of fuzz cells from the epidermal cell layer of seed coat. The DEGs identified in this study based on transcriptome sequencing suggest fuzz initiation involves diverse pathways related to cell wall modifications and integrity that are important for amino acid transport, Ca^2+^-signaling, and polarized cell wall outgrowth. Many TFs, cell wall loosening enzymes, and genes with an unknown function seem to be crucial components of these pathways. Overall, the results presented in this study indicate that the *N_5_* locus plays an indispensable role in fuzz initiation, probably by regulating accumulation of amino acids, Ca^2+^-signaling, and synthesis and deposition of cell wall loosening enzymes. The *N_5_* locus may achieve these regulatory roles directly or indirectly through TFs regulating these biological processes.
